# The SRP signal sequence of KdpD

**DOI:** 10.1038/s41598-019-45233-9

**Published:** 2019-06-18

**Authors:** Eva Pross, Andreas Kuhn

**Affiliations:** Institute of Microbiology, University of Hohenheim, 70599 Stuttgart, Germany

**Keywords:** Cellular microbiology, Chaperones

## Abstract

KdpD is a four-spanning membrane protein that has two large cytoplasmic domains at the amino- and at the carboxyterminus, respectively. During its biogenesis KdpD binds to the signal recognition particle (SRP) of *Escherichia coli* that consists of a 48-kDa protein Ffh and a 4.5S RNA. The protein is targeted to the inner membrane surface and is released after contacting the SRP receptor protein FtsY. The information within the KdpD protein that confers SRP interaction was found in the amino-terminal cytoplasmic domain of KdpD, particularly at residues 22–48. Within this sequence a Walker A motif is present at residues 30–38. To determine the actual sequence specificity to SRP, a collection of mutants was constructed. When the KdpD peptides (residues 22–48) were fused to sfGFP the targeting to the membrane was observed by fluorescence microscopy. Further, nascent chains of KdpD bound to ribosomes were purified and their binding to SRP was analysed by microscale thermophoresis. We found that the amino acid residues R22, K24 and K26 are important for SRP interaction, whereas the residues G30, G34 and G36, essential for a functional Walker A motif, can be replaced with alanines without affecting the affinity to SRP-FtsY and membrane targeting.

## Introduction

In *Escherichia coli*, the cytoplasmic signal recognition particle (SRP) mediates co-translational targeting of membrane proteins by binding to a “signal sequence” and generating a ribosome nascent chain complex (RNC)^1,2^. The RNC then binds to a membrane-associated SRP receptor, FtsY for membrane targeting^3^. SRP is universally conserved in its core region that consists of a ribonucleoprotein particle, the SRP RNA (4.5S RNA in *E. coli*) and the protein component SRP54 (Ffh in *E. coli* for “fifty-four-homolog”) that binds to the conserved RNA domain IV^4^. However, the composition of SRP varies among the different organisms with the most evolved version found in eukaryotes^5^. In contrast to the signal sequences of exported proteins, the bacterial SRP signal sequences are more hydrophobic and are mostly “uncleaved signal sequences” present in membrane proteins that remain in the final protein-chain as transmembrane anchor sequences. In general, the signal for insertion into the inner bacterial membrane is located in the first hydrophobic transmembrane domain and insertion is catalysed by the Sec translocase and/or YidC insertase.

SRP is bound to the ribosome and is ready to interact with a nascent protein chain. The amino-terminal NG domain of SRP is bound to the ribosomal proteins uL23 and uL29, next to the tunnel exit and the carboxy-terminal M domain to the ribosomal 23S RNA^6^. SRP at the ribosomal exit tunnel scans a nascent chain for bearing a hydrophobic SRP signal sequence^7^. The presence of such an emerging SRP signal sequence causes a tight binding to the hydrophobic groove of the SRP M domain^8^. This RNC-SRP complex then interacts with the membrane-associated SRP receptor, FtsY^3^. There, SRP and FtsY engage into a tight complex^3,9^ which results in the coordinated activation of the SRP/FtsY GTPase activities in Ffh and in FtsY at the membrane, essential for protein translocation^10,11^. After GTP hydrolysis, the RNC-SRP-FtsY complex is dissociated and the nascent chain is then released^12^ to further interact with the membrane, the Sec translocase or the YidC insertase^13,14^. In addition, SRP and FtsY are programmed for the next targeting cycle.

In this “SRP pathway”, an open question is how a signal sequence is recognized since no consensus motif exists and is relevant for SRP binding. With cross-linking studies, it could be shown that the hydrophobicity of the signal sequence is crucial for SRP binding, since lowering the hydrophobicity resulted in less efficient cross-linking^15,16^. The increase of the hydrophobicity in the signal sequences of *E. coli* presecretory proteins makes it possible to re-route the SecA dependent preproteins into the SRP targeting cycle^17^. Furthermore, it is suggested that the binding of SRP to the signal sequence is promoted by the presence of basic amino acid residues through electrostatic interactions^18^. The methionine-rich M domain of the Ffh protein binds hydrophobic residues of the substrate protein and accommodates the SRP-signal sequence in a hydrophobic groove^19^. A high resolution structure of Ffh from *E. coli* with a bound signal sequence showed that it was bound to the M-domain^6^. The structure shows that the signal sequence is sandwiched between the αM1 and αM4 helices of the Ffh protein and a hairpin loop of the ribosomal protein uL24. This binding involves both, hydrophobic- and electrostatic-mediated contacts.

Some inner membrane proteins have a large N-terminal cytoplasmic domain, like the sensor protein KdpD with the first transmembrane segment (TMS) starting at amino acid position 400. Previous data have suggested that KdpD has its signal sequence in the cytoplasmic domain, in a short amphiphilic sequence (aa 22–48) that targets the SRP complex to the inner membrane^20^. After targeting, the amphiphilic sequence is released from SRP and folds into an ATP binding domain. During the ongoing translation the transmembrane segments of KdpD are exiting the ribosome and can readily insert into the membrane even if YidC or SecYEG have been depleted in cells^21^. The KdpD signal sequence contains five positively charged residues, in which three are closely spaced (aa 22–26) and a stretch of 10 hydrophobic residues (aa 27–36) which is too short to span the membrane. The peptide contains also a Walker A motif, which is similar to classical ATP binding sites^22,23^. In the present study, we investigated in detail the involvement of the Walker A motif and the positively charged residues in the signal sequence binding to SRP and the membrane targeting using sfGFP fusion proteins. We found that when the glycine residues at positions 30, 34 and 36 are replaced by alanines the sfGFP fusion protein was still targeted to the membrane surface although these mutations destroy the Walker Box. In contrast, when the positively charged residues outside the Walker box at positions 22, 24 and 26 are replaced by glutamines membrane targeting was inhibited.

## Results

### Membrane targeting of N22-48-sfGFP depends on SRP

**Figure 1:**
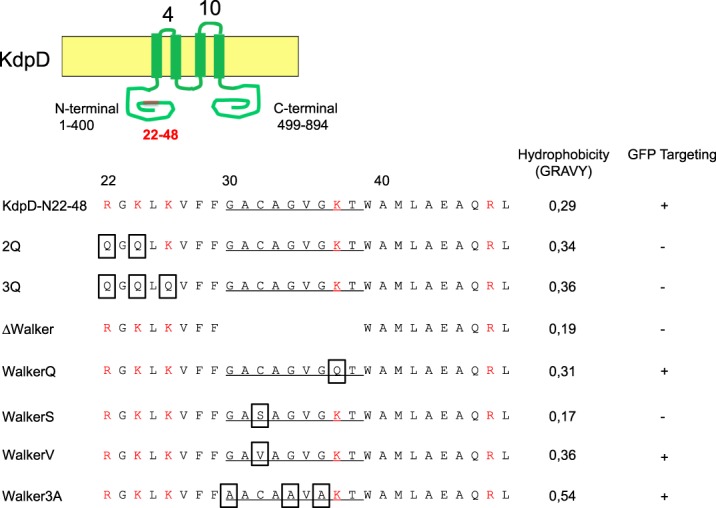
Topology of KdpD and the amino acid sequence of the N-terminal region (aa 22–48) of KdpD that is involved in SRP interaction. The KdpD protein is a four-spanning membrane protein with two large cytoplasmic domains at the N-terminus and the C-terminus. The SRP signal peptide of KdpD is located in the N-terminal domain at residues 22–48 and contains five positively charged residues (red letters) and a Walker A motif (underlined). The mutations studied here are marked with a box. Site-directed mutagenesis was used to alter the amino acids in the peptide. The hydrophobicity of each mutant is indicated.

**Figure 2:**
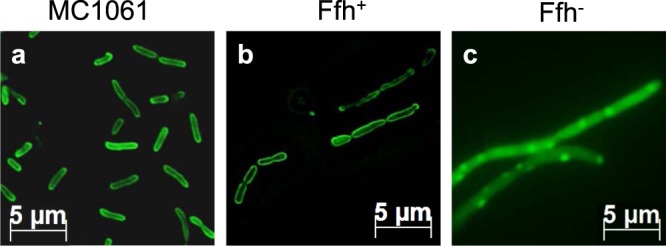
Membrane targeting of the KdpD signal peptide depends on SRP. Expression of N22-48-sfGFP in *E. coli* MC1061 (**a**) and in the depletion strain MCΔFfh (**b**,**c**) was induced for 30 min (**a**) and 20 min (**b**,**c**) at 37 °C and followed by fluorescence microscopy. Under arabinose growth conditions Ffh is present in the cells and allow the N22-48-GFP fusion protein to target to the membrane surface (**b**). Under glucose conditions, Ffh is depleted and the fluorescence signal of N22-48-sfGFP is patchy or distributed throughout the cytoplasm (**c**). Non-fused sfGFP was always distributed throughout the cytoplasm, regardless whether Ffh was present or not (Fig. S1).

The integral sensor protein KdpD consists of two large cytoplasmic domains, located at the N- and C-terminus (1–400 and 499–894), which are separated by four closely spaced transmembrane segments (401–498). Recently, it was shown that the amino acid residues 22–48 at the beginning of the N-terminal cytoplasmic region of KdpD (Fig. 1) serve as the SRP signal sequence^20^. In order to visualize the localization of N22-48 of KdpD we fused the N-terminal peptide (22–48) to sfGFP. To verify the involvement of SRP in the membrane targeting of the N22-48-sfGFP, we now analysed the localization of the N22-48-sfGFP in the Ffh-depletion strain MC∆Ffh and found that SRP is required for membrane targeting of N22-48-sfGFP. The MC1061 cells expressing N22-48-sfGFP were examined under the fluorescence microscope and found at the inner membrane (Fig. 2a). Likewise, when the MC∆Ffh cells were grown in presence of arabinose to express Ffh we also found the fusion protein at the surface of the inner membrane (panel b). However, when the cells were grown without arabinose and did not express Ffh (panel c, Fig. S3) most of the fluorescent signal appeared as punctuates in the cells that are indicative of aggregate formation. For controls, the sfGFP without the KdpD peptide remained in the cytoplasm under all the conditions (Fig. S1).

### SRP signal mutants of KdpD-N22-48-sfGFP

Mutants were generated to explore the function of the N22-48 sequence listed in Fig. 1. The positively charged residues at 22, 24 and 26 were substituted with glutamyl residues giving the 2Q and 3Q mutants. In addition to hydrophobicity, it is assumed that the presence of basic amino acid residues promote signal sequence binding to SRP^18^. The KdpD signal element contains a Walker A motif (residues 30 to 38), which is very similar to the classical ATP binding site. We constructed a mutant where the entire Walker box element was deleted (∆Walker). The influence of the residues within the Walker box on SRP-dependent membrane targeting of KdpD was investigated by a number of mutants generated in the Walker motif. The conserved lysyl residue in the Walker box was exchanged with a glutamyl in the WalkerQ mutant, the cysteine at residue 32 was substituted with a serine or valine and the conserved glycines at 30, 34 and 36 were exchanged with alanines in the Walker3A mutant. All mutant proteins were expressed in *E. coli* MC1061 for 30 min and analysed by SDS-PAGE (Fig. S2).

**Figure 3:**
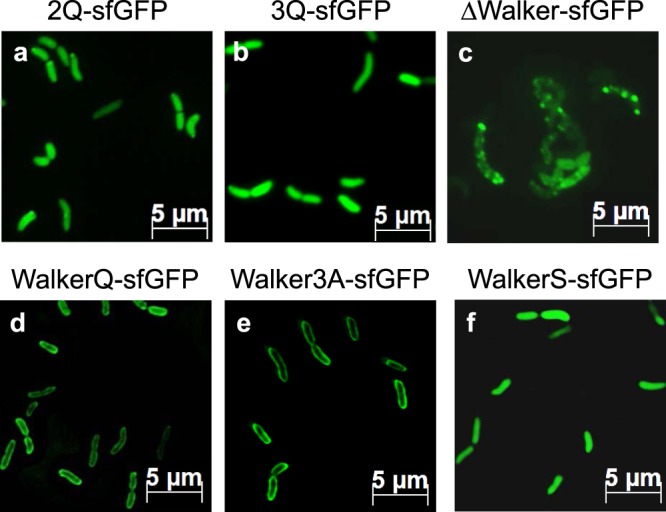
Targeting of the KdpD-GFP mutants. The KdpD-sfGFP fusion mutants 2Q (**a**) and 3Q (**b**) have 2 or 3 positively charged residues mutated to glutamines, respectively. The KdpD-sfGFP fusion mutant ΔWalker (**c**) and WalkerQ (**d**) have all residues of the Walker box deleted, WalkerQ has the conserved lysine residue 37 mutated into glutamine (**d**), Walker3A has the 3 glycine residues at residues 30, 34 and 36 mutated to alanines (**e**) and WalkerS has the cysteine residue 32 mutated to a serine (**f**), respectively. They were expressed in *E. coli* MC1061 at 37 °C and induced with 1 mM IPTG for 30 min. Except for the WalkerQ and the Walker3A mutant, the fluorescent signal was found distributed throughout the cytoplasm or in patches.

To analyse the effects of substituting the positively charged residues of 2Q and 3Q, the sfGFP fusion proteins were expressed in MC1061 (Fig. 3). The cells were grown to a density of 0.5 at OD_600_, induced with 1 mM IPTG and grown for 30 min at 37 °C. The cells were then applied for the fluorescence microscopy. In contrast to KdpD22-48 the fluorescence of the 2Q and 3Q proteins was detected uniformly in the cytoplasm (a, b). This shows for both mutants that the membrane targeting of the sfGFP fusion proteins was clearly inhibited. We conclude that the positively charged residues in the SRP signal are essential to target sfGFP to the membrane surface.

The residues 30 to 38 resemble the Walker motif. When these residues were deleted, the cells accumulated the sfGFP fusion protein in large aggregates at the poles of the cells (Fig. 3c). With the WalkerQ mutant where the conserved lysyl residue was substituted by a glutamyl we observed a clear fluorescence signal at the membrane (Fig. 3d).

**Figure 4:**
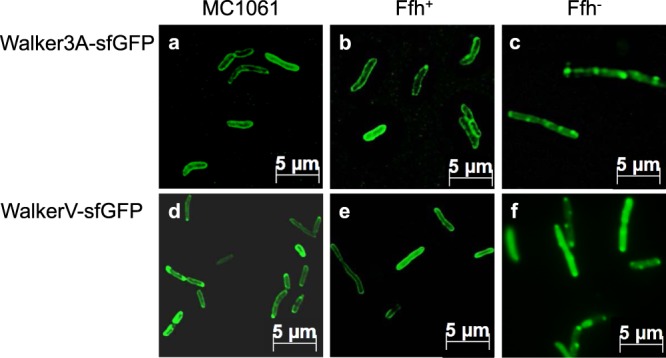
SRP-dependence of the functional Walker mutants. Localization of the Walker3A-sfGFP (**a**–**c**) and the WalkerV-sfGFP (**d**–**f**) mutant proteins in *E. coli* MC1061 (**a**,**d**) and MCΔFfh (**b**,**c**,**e**,**f**) after induction with 1 mM IPTG for 30 min (**a**,**d**) and 20 min (**b**,**c**,**e**,**f**) at 37 °C. When the cells were grown to express Ffh the fusion protein was localized at the membrane, whereas after depletion of Ffh (**c**,**f**) the fluorescence signal was found patchy at the cell poles or distributed in the cytoplasm. The depletion of Ffh was verified by Western blot (Fig. S3). The mutation of cysteine in valine at position 32 restores the targeting defect of the WalkerS mutant in *E. coli* MC1061 (Fig. 3f), where the cysteine residue was mutated into serine.

Next, the function of the glycyl residues in the Walker box was investigated. The Walker3A mutant with the conserved glycines exchanged to alanines was expressed in MC1061 (Figs 3e, 4a) and in MC∆Ffh cells (Fig. 4b,c). When Ffh is present as is in MC1061 (Fig. 4a) and when the MC∆Ffh cells were grown with arabinose (panel b), the fluorescence was found at the membrane indicating that the mutant protein was clearly targeted to the membrane. The targeting of this mutant was also sensitive to the depletion of Ffh (panel c). We conclude that the WalkerQ and the Walker3A mutants are fully functional to interact with SRP and are targeted to the membrane surface. When SRP is depleted the targeting of these mutants was inhibited similar to the wild-type sequence (Fig. 2).

When the cysteine residue at 32 in the Walker box was replaced by a serine the targeting to the membrane was inhibited (Fig. 3f). This might be because the hydrophobicity of the signal sequence is reduced (Fig. 1). Replacing the serine residue by the more hydrophobic residue valine restored the membrane targeting of the mutant (Fig. 4d). Also, the targeting of the WalkerV mutant was still sensitive to the depletion of Ffh (panel f). We conclude that not the cysteine residue at position 32 is important for membrane targeting but the hydrophobicity since mutation to valine with a comparable hydrophobicity restored the membrane targeting defect of the WalkerS mutant.

### Binding of KdpD22-48 to the hydrophobic groove of SRP

**Figure 5:**
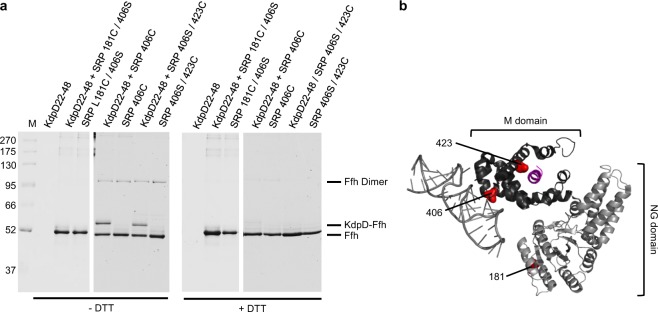
*In vitro* cross-linking of KdpD22-48 with SRP. (**a**) Copper phenanthroline cross-linking of the *in vitro* synthesized KdpD22-48 peptide with wildtype Ffh (406C), Ffh 181C/C406S or Ffh 406S/423C. 2 µM of reconstituted SRP was incubated with 20 µM of the peptide and 1 mM copper phenanthroline for 1 h on ice. Samples were TCA precipitated, resuspended in buffer with or without DTT and analysed by SDS-PAGE. The crosslinked peptide to Ffh 406C and 423C in the M domain generated a shifted band (KdpD-Ffh) but not for the 181C mutant in the G domain of SRP. For controls, the peptide and the proteins alone were analyzed. (**b**) The crystal structure of *E. coli* SRP (Pdb: 5GAD, the ribosome and FtsY are not shown for clarity) is displayed in grey, the signal sequence in purple and the positions of the cysteine residues are highlighted as red dots.

To explore whether the SRP signal sequence of KdpD is recognized by the hydrophobic groove formed by the M domain of SRP, cross-linking experiments with purified SRP and synthesized KdpD peptides 22–48 were performed. Since the wildtype Ffh has a cysteine residue at position 406 in the M domain disulfide cross-linking was tested with the *in vitro* synthesized KdpD22–48 peptide that has a cysteine residue at position 32. In addition, two mutant Ffh proteins with a cysteine residue at position 181 in the G domain or at 423 in the M domain pocket were analysed. Both mutants were combined with a serine at 406. After mixing the proteins cross-linking with copper phenanthroline was performed for 1 h on ice. The samples were analysed on an SDS-PAGE with and without DTT, respectively (Fig. 5). When the KdpD peptide was incubated with the wildtype SRP having a cysteine residue in the M domain or at position 423 in the M domain an additional band appeared indicating that the peptide was cross-linked to SRP (lane 5, 7). The cross-link was sensitive to the reducing agent DTT (Fig. 5, lane 12, 14). The shifted band did not appear when KdpD or SRP alone was incubated with the cross-linking agent. In contrast to SRP and SRP 423, the addition of the copper phenanthroline to the KdpD peptide with SRP L181C (in the G domain) showed no cross-linked band (lane 3). In conclusion, our results are consistent with the KdpD signal sequence binding in the same position of SRP as other and canonical signal sequences do^6,24^.

### Binding of the KdpD mutants to SRP

To test whether the observed membrane targeting abilities of the mutants correlate with binding capabilities to SRP we analysed the interaction of the 22–48 peptide with the wild-type sequence, the 3Q mutant and the Walker3A mutant with purified SRP employing microscale thermophoresis (MST).

**Figure 6:**
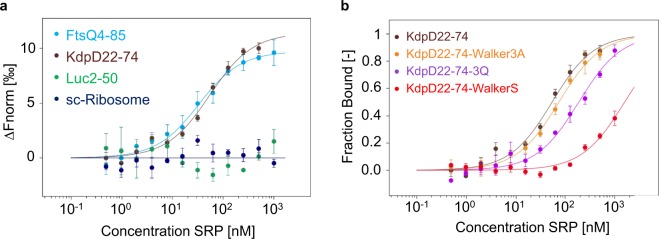
Binding of ribosome nascent chains (RNC) to SRP. Microscale thermophoresis (MST) measurements of unlabeled SRP (1 µM to 0.49 nM) with labeled RNCs (5 nM) of (**a**) FtsQ4-85 (blue dots), KdpD22-74 (brown dots), Luc2-50 (green dots), short-chain-ribosomes (dark blue dots) and (**b**) with KdpD22-74-Walker3A (orange dots), KdpD22-74-3Q (purple dots), KdpD22-74-WalkerS (red dots). After a 5 min incubation on ice the dilutions were filled into Premium capillaries (NanoTemper Technologies) for the MST measurements. In (**a**), the change in normalized fluorescence (∆Fnorm in ‰) and in (**b**), the fraction bound, is plotted against the concentration of unlabeled SRP (3 independently pipetted measurements, error bars represent the standard deviation). The raw data of the measurements are shown in Figs S4 and S5.

Ribosome-nascent chains (RNC) were designed by introducing the sequence for KdpD22-74 or the mutant sequences in a TnaC stalling plasmid resulting in tryptophan dependent ribosome stalling. Since there are about 30 amino acids in the ribosomal exit tunnel the KdpD peptide 22–48 was expected to be exposed outside the tunnel, so that the accessibility is guaranteed. The different his-tagged RNCs were purified and labeled with the fluorescent dye NT-647. The interaction studies of RNCs and purified SRP were analyzed using microscale thermophoresis with a fixed concentration of 5 nM for the labeled RNCs and varying concentrations of 1 µM to 0.5 nM for unlabeled SRP. First, RNCs encoding 22–74 as nascent chain were incubated with SRP resulting in a binding event comparable with the positive control, where RNCs with amino acids 4–85 of the SRP substrate FtsQ as nascent chain were used (Fig. 6a). As a negative control, ribosomes with amino acids 2–50 of the cytoplasmic protein firefly luciferase as a nascent chain were incubated with SRP, which showed no binding in this concentration range (a). In addition, RNCs with only a short nascent chain of about 13 amino acids, mimicking ribosomes at the very beginning of translation, were used as an additional control. Like for Luc2-50-RNCs, also these short-chain ribosomes showed no binding to SRP under these conditions (a). The exchange of three conserved glycines in alanines in the Walker A motif in the KdpD nascent chain did not affect the SRP binding and was similar to that of KdpD22–74 (Fig. 6b). In contrast, the exchange of three positively charged amino acids at position 22, 24 and 26 resulted in a slightly different binding indicating a reduced affinity to SRP. The exchange of the cysteine at position 32 in the Walker A motif to a serine residue led to a very weak SRP binding resulting in the fact that no saturation could be reached in the measured concentration range. We conclude that both mutants that were inhibited for membrane targeting were affected in binding. Therefore, the positively charged N-terminal part of the peptide and the hydrophobicity of the core region are critical for SRP binding.

### Binding of the KdpD mutants to SRP-FtsY

**Figure 7:**
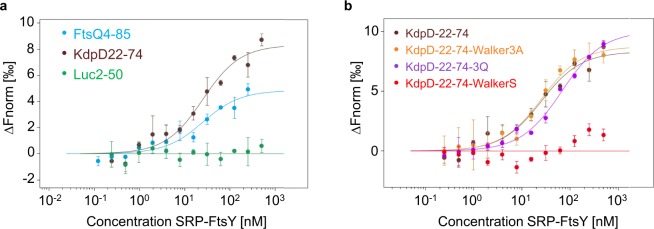
Binding of ribosome nascent chains (RNC) to SRP-FtsY. Microscale thermophoresis (MST) measurements of unlabeled SRP-FtsY complex (500/250 nM to 0.25/0.12 nM) with labeled RNCs (5 nM) of (**a**) FtsQ4-85 (blue dots), KdpD22-74 (brown dots), Luc2-50 (green dots) and (**b**) with KdpD2274-Walker3A (orange dots), KdpD22-74-3Q (purple dots), KdpD22-74-WalkerS (red dots). After a 5 min incubation on ice the dilutions were filled into Premium capillaries (NanoTemper Technologies) for the MST measurements. The change in normalized fluorescence (∆Fnorm in ‰) is plotted against the concentration of the unlabeled SRP-FtsY complex (3 independently pipetted measurements, error bars represent the standard deviation). The raw data of the measurements are shown in Figs S6 and S7.

After the SRP-dependent membrane targeting the SRP-nascent-chain complex is expected to interact with the SRP receptor FtsY. The binding of FtsY to SRP results in conformational changes leading to a stronger binding of SRP to the nascent chain^10^. This only occurs if SRP is bound to a correct cargo. Therefore, we analyzed whether the different KdpD-nascent chains are able to bind to a closed SRP-FtsY complex containing GTP. For the MST binding experiments, the reconstituted SRP was incubated with purified FtsY in a molar ratio of 1:4 in presence of 200 µM of the non-hydrolysable GTP analogue GppNHp. MST experiments were done with a fixed concentration of 5 nM for the labeled RNCs and varying concentrations of 250 nM/500 nM to 0.12/0.24 nM for unlabeled SRP-FtsY complex. First, RNCs with amino acids 22–74 of KdpD as a nascent chain were incubated with the closed SRP-FtsY complex. The MST measurements showed that the SRP-FtsY complex is able to bind to the RNCs exposing residues 22–48 of KdpD (Fig. 7a). In contrast, the negative control with residues 2–50 of firefly luciferase showed no binding with the SRP-FtsY complex under these conditions (Fig. 7a). From this we conclude that RNCs with residues 22–74 of KdpD represent a correct cargo for SRP. Also, the interaction with KdpD-RNCs where the three conserved glycines in the Walker A motif were mutated in alanines showed an efficient binding to the SRP-FtsY complex (Fig. 7b). Thus, the exchange of the glycines to alanines known to be essential for the function of the Walker motif did not affect SRP-FtsY binding. In contrast, the mutant where the three positively charged amino acids at position 22, 24 and 26 were substituted with glutamines showed a weaker binding to the SRP-FtsY complex as was with only SRP (Fig. 7b). Since even in this case binding to a closed SRP-FtsY complex was observed we conclude that SRP-FtsY binding is not inhibited but is weakend after the exchange of the three positively charged amino acids. The WalkerS mutant with the lowest hydrophobicity (GRAVY = 0.17; GRAVY 22–48 = 0.29) showed the weakest binding to SRP. Therefore, the substitution of the cysteine residue with a serine residue prevents binding of the SRP-FtsY complex (Fig. 7b). Taken together, our experiments show that both, the positively charged N-terminal part of the sequence and the hydrophobicity of the core region are critical for SRP binding and, in addition, for FtsY recruitment.

## Discussion

The amino-terminal region of the KdpD sensor protein has two functions. In the fully folded protein it binds ATP that might modulate the communication of KdpD with its response regulator protein KdpE^23^. ATP is bound by a classical Walker box where the Walker A motif is located at the residues 30 to 38 and a potential Walker B motif at residues 105 to 110. The second function of the amino-terminal region is early during its synthesis in the ribosome. For the targeting to the membrane the amino-terminal region of KdpD at the residues 22–48 is first contacting the SRP^20^. The RNC-SRP complex is then transported to the receptor protein FtsY to ensure that the nascent protein chain is close to the membrane surface. We show here that an artificially stalled KdpD22-74 RNC binds to SRP comparable with RNCs exposing the positive control protein FtsQ4–85, a well-studied substrate of SRP (Fig. 6)^25^. In addition, also a preassembled SRP-FtsY complex which was activated by GTP could be bound to KdpD22-74 RNC as for FtsQ4-85 RNC (Fig. 7). These results show that the residues 22–48 efficiently bind to SRP and allow the nascent chain of KdpD an early targeting to the membrane surface before translation has synthesized the full 894 long protein. The binding to SRP was analysed with microscale thermophoresis. Fluorescently labeled RNCs at 5 nM were mixed 1:1 with purified and assembled SRP (Ffh and 4.5 RNA) in different dilutions (0.5 nM to 1 μΜ) and applied to capillaries. For the binding to SRP-FtsY the assembled SRP was mixed with purified FtsY and 200 μΜ GppNHp prior to the addition of RNCs. Previous affinity determinations were performed with fluorescence equilibrium titrations using fluorescence-labeled SRP with leader peptidase RNCs^26^ or by fluorescence anisotropy of fluorescein-labeled SRP with EspP RNCs^27^ resulted in comparable binding events. Since the SRP-bound cargo has to pass a number of checkpoints, the binding to a preassembled SRP-FtsY complex with high affinity is an important step in the pathway^27^.

The early targeting of KdpD at the membrane surface ensures that the 4 transmembrane segments can readily insert when they appear at the ribosome tunnel exit^28^. Previous studies have shown that the insertion of KdpD into the inner membrane can occur even in the absence of SecYEG or YidC^21^. Most likely, the cotranslational membrane insertion directly into the bilayer is possible because the periplasmic loops are very short with 4 and 10 residues, respectively (Fig. 1).

C-tailed membrane proteins share with KdpD the fact that a large hydrophilic domain is released from the ribosome before a membrane anchoring segments is exposed at the tunnel exit. We have recently shown that the membrane targeting and insertion of the C-tail protein SciP involves SRP and the SRP receptor^29^. Similar as in KdpD the TMS of SciP is far from the N-terminus (residues 184–206). We found that two short hydrophobic regions at residues 12–20 and 62–71 of SciP have the potential to interact with SRP. When these peptides were fused with sfGFP the fusion protein was targeted the membrane surface. We conclude that the amino-terminal sequences found in SciP and KdpD are functional SRP signal sequences and are located close to the N-terminus in membrane proteins that contain large cytoplasmic domains. In both cases, the SRP signal sequence is separate from the first membrane spanning segment and therefore allows an early binding of SRP before the membrane segments are translated by the ribosome.

The function of the SRP-signal sequence can be tested in a fusion protein with the green fluorescent protein GFP^20^. Here, we show that the assay can be improved when the super folding GFP (sfGFP) is used for the fusion. The fluorescence in cells can be observed already 20 min after induction. With this assay we tested a collection of mutants that affected the function of the 22–48 sequence as a SRP signal and/or Walker A element. The key lysine of the Walker A element cannot be replaced by another amino acid without affecting the function of the Walker element^30^. When we substituted the key lysine with a glutamine in the WalkerQ mutant, or when the conserved glycine residues at position 30, 34 and 36 in the Walker3A mutant were replaced by alanine residues, membrane localization was not affected (Fig. 3d,e). Likewise, the binding events of the Walker3A mutant to SRP and to SRP-FtsY corresponded to the binding we had obtained with the wild-type signal sequence of 22–48 (Figs 6, 7). Therefore, these modified sequences fully function as a SRP targeting signal.

The deletion of the Walker A element (residues 30–38) resulted in a patchy appearance of the fusion protein in the cells (Fig. 3c). A similar phenotype was observed for the wild-type sequence when Ffh was depleted (Fig. 2c). We assume that the patches form because of aggregation in the cytoplasm. Interestingly, the sfGFP itself did not form such aggregates, even when Ffh was depleted (Fig. S1). It is surprising that small 19 to 28 residues long N-terminal extensions of sfGFP have such a big effect on the solubility. The binding affinity of the WalkerS mutant to SRP was the lowest we had measured and there was no binding to SRP-FtsY. It is possible that the lower hydrophobicity of the cysteine is critical in allowing that the sequence is still able to bind to SRP. The malfunctioning of this mutant in membrane targeting was supported by the fluorescence microscopy (Fig. 3f). Interestingly, mutation of the cysteine residue into the more hydrophobic valine restored membrane targeting indicating that not the cysteine residue but the hydrophobicity is critical for SRP binding (Fig. 4d).

Finally, the positively charged residues at 22, 24 and 26 were investigated for membrane targeting and SRP binding. The sfGFP fusion protein showed that the fluorescence was evenly distributed in the cytoplasm suggesting that the binding to SRP is affected. Indeed, the binding to SRP and to SRP-FtsY was lower compared to the wild-type signal sequence of 22–48 respectively (Figs 6, 7). This shows that the positively charged residues play an important role for the interaction with SRP.

Taken together, this study shows that a SRP signal sequence is not restricted to a transmembrane segment but can be localized in a cytoplasmic region. The results obtained with the mutants of the signal sequence underline the importance of the positively charged N-terminal part and the hydrophobic C-terminal part of the signal sequence to allow the interaction with the M-pocket of SRP and membrane targeting.

## Methods

### Bacterial strains and culture conditions

*E. coli* MC1061 *(hsdR mcrB araD139 (ΔaraABC-leu)7697 lacX74 galU galK rspL thi*) was decribed^31^. For Ffh depletion, *E. coli* MC∆Ffh^32^ was grown overnight in LB medium containing 0.2% (w/v) arabinose and 0.4% (w/v) glucose, washed in medium lacking arabinose and back-diluted 1:100 in LB medium containing 0.4% (w/v) glucose. Media preparation and bacterial manipulations were performed according to standard methods^33^. Where appropriate, ampicillin (100 µg/mL, final concentration) was added to the medium. *E. coli* BL21 (DE3)^34^ and *E. coli* KC6^35^ were grown overnight in LB with ampicillin (100 µg/mL) and back diluted 1:100 in fresh LB with ampicillin (100 µg/mL).

### Construction of SRP-signal mutants of KdpD-N22-48-sfGFP

All oligonucleotides used in this study are listed in the Supplementary Table S1 and the used plasmids in the Supplementary Table S2. The amino acid residues 22 to 48 of KdpD were amplified flanking the PCR product with a *Hind*III and *Bam*HI recognition site. The PCR product was then cloned in a pMS119EH derivate containing the *sfGFP* gene (own collection) using the restriction enzymes *Hind*III and *Bam*HI. The different SRP-signal mutants of KdpD-N22-48-sfGFP were constructed by a PCR-based mutagenesis of N22-48-sfGFP. Substitution of the basic residues at position 22 and 24 of N22-48-sfGFP into glutamine resulted in the mutant pMS-KdpD22-48-2Q-sfGFP (named 2Q). Substitution of three of the closely spaced basic amino acid residues in N22-48-sfGFP were substituted into glutamines resulted in the mutant pMS-KdpD22-48-3Q-sfGFP (named 3Q). Substitution of lysine 37 within the Walker A motif with a glutamine resulted in mutant pMS-KdpD22-48-WalkerQ-sfGFP (named WalkerQ). To construct mutant pMS-KdpD22-48-∆Walker-sfGFP (named ∆Walker), amino acids 30–38 were deleted with site-directed mutagenesis. To change the conserved motif of the Walker box into a no Walker-motif a site-directed mutagenesis was done to generate pMS-KdpD22-48-Walker3A-sfGFP (named Walker3A). Substitution of cysteine 32 within the Walker A motif with a serine or valine resulted in mutant pMS-KdpD22-48-WalkerS-sfGFP (named WalkerS) and mutant pMS-KdpD22-48-WalkerV-sfGFP (named WalkerV). The coding regions of all constructs were verified by DNA sequence analysis.

### Construction of plasmids for ribosome-nascent-chain (RNC) interaction studies

For cloning of the TnaC-stalling sequence, pBAT4-MscL^115^ (kindly provided by R. Beckmann, Munich) was digested with *Xba*I and *Hind*III and cloned into pMS119EH^36^. To remove the MscL sequence a *Nco*I and *Mfe*I recognition site was introduced with site-directed mutagenesis resulting in pMS-MscL^115^-TnaC. The sequence encoding for amino acids 22–74 of KdpD was amplified flanking the PCR product with a *Mfe*I and *Nco*I recognition site. The digested PCR product was cloned into pMS-MscL^115^-TnaC using *Nco*I and *Mfe*I (pMS-KdpD22-74-TnaC). For cloning of pMS-KdpD22-74-W3A-TnaC and pMS-KdpD22-74-3Q-TnaC the sequence was amplified using the respective primers and template DNA and cloned into pMS-MscL^115^-TnaC using *Nco*I and *Mfe*I. Plasmid pMS-KdpD22-74-C32S-TnaC was generated by site-directed mutagenesis on plasmid pMS-KdpD22-74-TnaC. The plasmid pMS-His-HA-TnaC (named short-chain (sc)-ribosome) was cloned by digesting a pMS-TnaC plasmid (institute collection) with *Mfe*I and *Pst*I, blunted with T4 DNA polymerase and religated. This results in RNCs with exposing a short nascent chain of about 13 amino acids.

The plasmid encoding the first 50 amino acids of the cytoplasmic protein firefly luciferase was generated by amplification from plasmid pUC19-T7-Luc^50^ (kindly provided by Shu-ou Shan; Caltech). The PCR product was flanked with an *Eco*RI and a *Nco*I recognition site producing compatible sticky ends with *Mfe*I and *Nco*I digested plasmid resulting in plasmid pMS-Luc2-50-TnaC. The plasmid encoding the first 85 amino acids of FtsQ (pEM36-3C) was kindly provided by R. Beckmann, Munich.

### Fluorescence microscopy

Strains were grown overnight at 37 °C, diluted in fresh LB medium and grown to an OD_600_ of 0.5. IPTG was then added to a final concentration of 1 mM. The cells were incubated for 20 min (MC∆Ffh) or 30 min (MC1061) at 37 °C under continuous shaking. The cells were treated as described^29^ and collected by centrifugation, washed twice with LB medium and resuspended in 2 mM EDTA, 50 mM Tris-HCl, pH 8.0. The cell suspension (3 µL) was applied to a polylysine-coated cover glass (Sigma-Aldrich) and examined immediately by fluorescence microscopy with the Zeiss AxioImager M1 fluorescence microscope. Emission was detected with filters specific for GFP. Analysis was done by using the AxioVision Software (Zeiss). The expression of the different KdpD22-48-sfGFP mutants was analysed on a 12% SDS-PAGE (after TCA precipitation with 10% TCA) and immunoblotting with an α-GFP and α-rabbit antibody.

### Purification of Ffh and FtsY

*E. coli* Ffh was purified essentially as described by Seitl^37^. Wild-type Ffh and the mutant L181C and M423C were expressed from pMS-Ffh-C-Strep, pMS-Ffh-L181C-C-Strep or pMS-Ffh-M423C-C-Strep in BL21 (DE3) cells. 2 L LB with 100 µg/mL ampicillin were inoculated 1:100 from an overnight culture and grown at 37 °C until an OD_600_ of 0.5. The cells were induced with 1 mM IPTG for 3 h at 37 °C, harvested, resuspended in buffer A_Ffh_ (20 mM Hepes pH 8, 350 mM NaCl, 10 mM MgCl_2_, 10 mM KCl, 10% glycerol) and lysed using the OneShot at 1.23 kbar. Before cell disruption 0.2 mM PMSF was added. The lysate was centrifuged 2x for 30 min at 20 000 g and the supernatant was loaded onto 3 mL *Strep*-Tactin matrix by gravity flow. After washing the matrix with 50 mL buffer W_Ffh_ (20 mM Hepes pH 8, 500 mM NaCl, 10 mM MgCl_2_, 100 mM KCl) the protein was eluted with 20 mL buffer E_Ffh_ (20 mM Hepes pH 8, 350 mM NaCl, 10 mM MgCl_2_, 10 mM KCl, 10% glycerol, 2.5 mM desthiobiotin) in 2 mL fractions. The elution fractions were further purified using the Äkta-purifier System on a Superdex 75 16/60 column in buffer GF_Ffh_ (20 mM Hepes pH 8, 200 mM NaCl, 10 mM MgCl_2_, 10 mM KCl, 10% glycerol).

*E. coli* FtsY was expressed from plasmid pTrc99-FtsY-His (kindly provided by HG Koch, Freiburg) in BL21 (DE3) cells. 2 L LB with 100 µg/mL ampicillin were inoculated with an overnight culture (1:100) and grown at 37 °C until an OD_600_ of 0.5. The culture was induced with 1 mM IPTG for 4 h at 37 °C, the cells were harvested and resuspended in buffer A_FtsY_ (50 mM HEPES pH 7.6, 1 M NH_4_Ac, 10 mM Mg(OAc)_2_, 10% glycerol, 1 mM DTT). After the addition of 0.2 mM PMSF the cells were lysed using the OneShot at 1.23 kbar, the lysate was centrifuged for 30 min at 4300 g and the supernatant was centrifuged in a Beckman Ti60 rotor for 1 h at 38 000 rpm. The supernatant was incubated with 2 mL Ni-NTA in Buffer A_FtsY_ + 30 mM imidazole for 1 h at 4 °C on a rotary wheel. The matrix was washed with 20 mL buffer W_FtsY_ (50 mM HEPES pH 7.6, 1 M NH_4_Ac, 10 mM Mg(OAc)_2_, 1 mM DTT, 30 mM imidazole) and the protein was eluted with 20 mL buffer E_FtsY_ (50 mM HEPES pH 7.6, 1 M NH_4_Ac, 10 mM Mg(OAc)_2_, 400 mM imidazole, 10% glycerol) in 2 mL fractions. The elution fractions were further purified using the Äkta-purifier System on a Superdex 200 16/60 column in buffer GF_FtsY_ (100 mM HEPES pH 7.6, 200 mM KOAc, 20 mM Mg(OAc)_2,_ 2 mM DTT, 10% glycerol).

### *In vitro* cross-linking with copper phenanthroline

The KdpD peptide (RGKLKVFFGACAGVGKTWAMLAEAQRL) was synthesized by the Custom peptide synthesis services from GENOSPHERE Biotechnologies (France) with N-terminal acetylation and C-terminal amidation and a purity of >95%. For the *in vitro* cross-linking experiment the plasmid pMS-Ffh-L181C-C-Strep was generated by site-directed mutagenesis on plasmid pMS-Ffh-C-Strep.

For *in vitro* cross-linking 2 µM of purified Ffh (406 C), Ffh L181C/406S or Ffh 406S/423 C were mixed with 3 µM of 4.5S RNA to get a functional SRP in buffer_SRP_ (20 mM HEPES pH 7.2, 50 mM KOAc, 5 mM Mg(OAc)_2_). Each of the reconstituted SRP was mixed with 20 µM of synthesized KdpD peptide in buffer_Crosslink_ (50 mM Tris pH 7.4, 150 mM NaCl, 10 mM MgCl_2_) and 1 mM copper phenanthroline was added. The mixture was incubated for 1 h on ice, TCA precipitated, resuspended in buffer with or without DTT (1 mM) and loaded on a 10% SDS-PAGE.

### Construction and purification of ribosome-nascent-chains (RNCs)

The plasmids pEM36-3C (encoding for a N-terminal His-Tag, a C3-protease cleavage site, the first 85 amino acids of FtsQ, a C-terminal HA-Tag and the TnaC-stalling sequence), pMS-Luc2-50-TnaC, pMS-KdpD22-74-TnaC, pMS-KdpD22-74-W3A-TnaC and pMS-KdpD22-74-3Q-TnaC were transformed in *E. coli* KC6^35^ cells. 2 L LB containing 100 µg/mL ampicillin were inoculated 1:100 from an overnight culture and grown until an OD_600_ = 0.5. For induction, 1 mM IPTG was added and the cells were grown for another hour at 37 °C. After harvesting, cells were resupended in buffer A_RNC_ (20 mM Hepes pH 7.2, 250 mM KOAc, 25 mM Mg(OAc)_2_, 2 mM L-tryptophane, 0.1% DDM), 0.2 mM PMSF was added and the cells were lysed using the OneShot at 1.23 kbar. The lysate was centrifuged at 31000 g for 20 min, the supernatant was loaded onto 750 mM sucrose in buffer A_RNC_ and the ribosomes were pelleted at 25 000 rpm for 20 h in a Beckman Ti45 rotor. The ribosomal pellet was resuspended in buffer A_RNC_ and loaded onto 3 mL Ni-NTA (blocked with 10 µg/mL *E. coli* tRNA for 1 h at 4 °C) and batched for 1 h at 4 °C on a rotary wheel. The matrix was washed with 30 mL Buffer B_RNC_ (50 mM Hepes pH 7.2, 500 mM KOAc, 25 mM MgCl_2_, 2 mM L-tryptophane, 0.1% DDM) and the His-tagged ribosomes were eluted with 3 mL buffer B_RNC_ + 150 mM imidazole and 3 mL buffer B_RNC_ with 300 mM imidazole in 1 mL fractions. The elution fractions were loaded onto a linear sucrose gradient (10–40% sucrose in buffer B_RNC_) and centrifuged at 30 000 rpm for 3.5 h in a Beckman SW40 rotor. The gradient was collected in 1 mL fractions, the ribosome containing fractions were identified measuring the absorbance at 260 nm, pooled and centrifuged at 40 000 rpm for 4 h in a Beckman Ti60 rotor. The ribosomal pellet was resuspended in buffer C_RNC_ (20 mM Hepes pH 7.2, 50 mM KOAc, 5 mM Mg(OAc)_2_, 2 mM L-tryptophane) and stored at −80 °C.

### *In vitro* 4.5S RNA synthesis and SRP reconstitution

To get a functional SRP the purified Ffh protein was reconstituted with the *in vitro* synthesized 4.5S RNA as described by Seitl^37^. Therefore, plasmid pUC18-4.5S RNA (kindly provided by Irmgard Sinning, Heidelberg) was used where the 4.5S RNA sequence was placed downstream of the T7 promoter. First, the plasmid was linearized with *Bam*HI and gel purified. For *in vitro* transcription with the HiScribe™ T7 High Yield RNA Synthesis Kit (NEB) 1 µg linearized plasmid DNA was used. After incubation for 16 h at 37 °C the 4.5S RNA was purified using the RNA Clean & Concentrator^TM^ −25 Kit (Zymo Research), analyzed on a 2% agarose gel in 1x Tris-borate-EDTA buffer and stored at −80 °C. Prior to the reconstitution, the RNA was heated to 75 °C for 2 min and chilled on ice for 1 min. Ffh and 1.5-fold molar excess of 4.5S RNA were mixed in MST-buffer (20 mM HEPES pH 7.2, 50 mM KOAc, 5 mM Mg(OAc)_2,_ 2 mM L-tryptophane, 0.05% Tween-20) and incubated for 10 min at 20 °C.

### Labeling of RNCs

The different RNCs were labeled with the cysteine-reactive fluorescent dye NT-647 and the RED-maleimide labeling kit (NanoTemper Technologies). The RNCs were adjusted to a concentration of 2 µM in 100 µL buffer C_RNC_ and mixed with 3-fold molar excess of the dye in 100 µL labeling buffer provided in the kit. After incubation for 30 min at RT in the dark the labeling reaction was purified to remove the free dye using the column provided in the kit. The concentration of the labeled RNCs after purification was calculated measuring the absorbance at 260 nm and they were stored at −80 °C.

### Microscale thermophoresis

For the interaction studies between the RNCs and SRP, the labeled RNCs were adjusted to 10 nM in MST buffer. With the reconstituted SRP a series of 1:1 dilutions was prepared in MST buffer with a concentration ranging from 1 µM to 0.49 nM. The ligand dilutions were mixed with one volume of labeled RNCs resulting in a RNC concentration of 5 nM. After incubation for 5 min on ice, the dilutions were filled in Monolith NT Premium Treated capillaries (NanoTemper Technologies) and measured using the Monolith NT.115 instrument. During measurement the temperature was kept constant at 22 °C. Thermophoresis was measured with 5 s laser off, 20–30 s laser on and 5 s laser off, a LED power of 40–60% and the MST Power “Low”. The data of three independently pipetted measurements were merged and analyzed using the software MO.Affinity Analysis v2.3 (NanoTemper Technologies) using the manual evaluation (Cold region start/end: −1 s/0 s; Hot region start/end: 5.01 s/10.08 s).

For the interaction studies between the RNCs and SRP-FtsY a preincubated closed SRP-FtsY complex was used. Therefore, the reconstituted SRP was mixed with 4-fold molar excess of FtsY in MST buffer containing 200 µM of the non-hydrolysable GTP-analogue GppNHp. After incubation for 10 min at 25 °C the stable SRP-FtsY complex was incubated on ice. For measurements a series of 1:1 dilutions was prepared in MST buffer with 200 µM GppNHp with a complex concentration ranging from 500 nM/250 nM to 0.25/0.12 nM. The ligand dilutions were mixed with one volume of labeled RNCs resulting in a RNC concentration of 5 nM. After incubation for 5 min on ice, the dilutions were filled in Monolith NT capillaries and measured using the Monolith NT.115 instrument. During measurement the temperature was kept constant at 22 °C. Thermophoresis was measured with 5 s laser off, 20–30 s laser on and 5 s laser off, a LED power of 60% and the MST Power „low”. The data of three independently pipetted measurements were merged and analyzed using the software MO.Affinity Analysis v2.3 (NanoTemper Technologies) using the manual evaluation (Cold region start/end: −1 s/0 s; Hot region start/end: 5.01 s/10.02 s).

## Supplementary information

Dataset 1

## Data Availability

All data generated or analysed during this study are included in this published article (and its Supplementary Information Files) or are available from the corresponding author on reasonable request.
