# Supplementary material for: The SRP signal sequence of KdpD
Source: Sci Rep. 2019 Jun 18;9:8717. doi: 10.1038/s41598-019-45233-9 (PMC6581901; doi:10.1038/s41598-019-45233-9)
Supplement: Supplementary file 1 — Dataset 1 [file 41598_2019_45233_MOESM1_ESM.docx]

## The SRP signal sequence of KdpD

Eva Pross and Andreas Kuhn*

Institute of Microbiology,

University of Hohenheim,

70599 Stuttgart, Germany

* Corresponding author

Tel.: 0049-711-459-22222

Fax: 0049-711-459-22238

E-mail: [andikuhn@uni-hohenheim.de](mailto:andikuhn@uni-hohenheim.de)

**Supplementary Information**

**Figure S1**


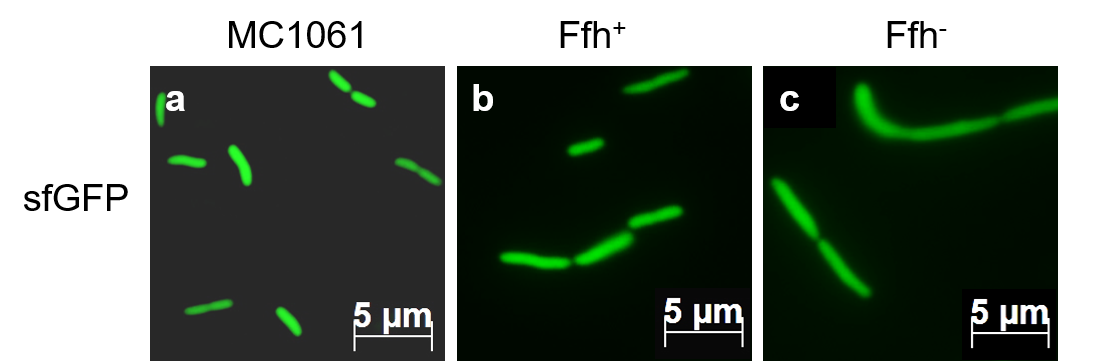


**Supplementary Figure S1: Cellular localization of sfGFP**

Expression of sfGFP in *E. coli* MC1061 (a) and in the depletion strain MCΔFfh (b, c) was induced for 30 min (a) and 20 min (b, c) at 37°C and followed by fluorescence microscopy. Non-fused sfGFP was always distributed throughout the cytoplasm, regardless whether Ffh was present or not.

**Figure S2**

**
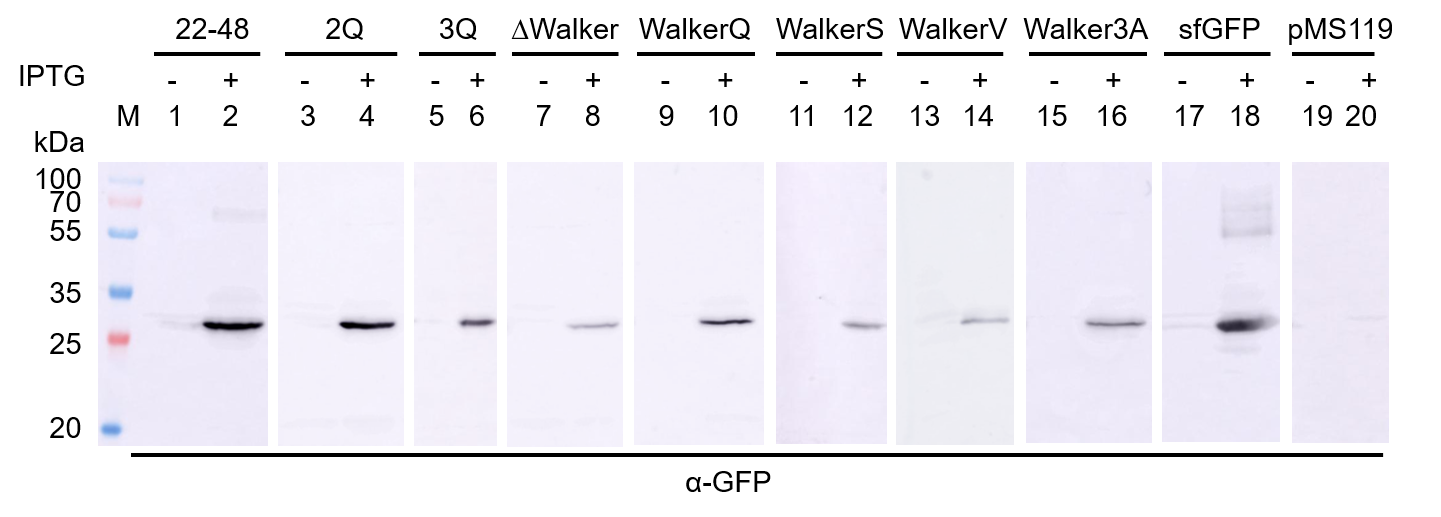
**

**Supplementary Figure S2: Expression of the different KdpD-sfGFP fusion proteins in *E. coli* MC1061**

Expression was induced at an OD_600_ of 0.5 for 30 min, the cells were TCA precipitated and loaded on a 12% SDS-PAGE. After Western transfer, immune detection was carried out with α-GFP and α-rabbit antibodies. Due to induction, a band between 25 and 35 kDa corresponding to the different KdpD-sfGFP fusion proteins and for the non-fused sfGFP was detected. The empty plasmid was analysed as a control.

**Figure S3**

**
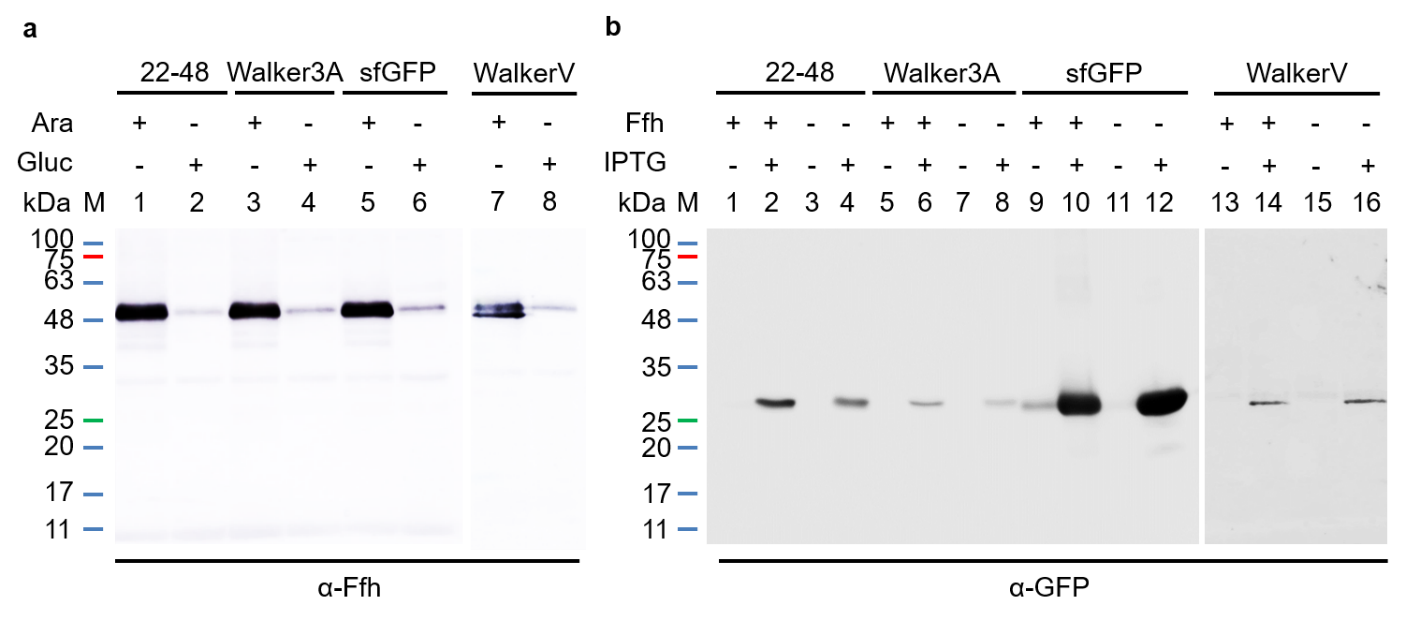
**

**Supplementary Figure S3: Expression of the different KdpD-sfGFP fusion proteins in *E. coli* MCΔFfh.**

(a) The amount of Ffh before the induction of N22-48-sfGFP, Walker3A-sfGFP, non-fused sfGFP and WalkerV-sfGFP under arabinose (0.2%) and glucose (0.4%) growth conditions. The amount of Ffh was verified by Western blot with α-Ffh and α-rabbit antibodies. Growth in glucose leads to depleted Ffh levels. (b) Expression of N22-48-sfGFP, Walker3A-sfGFP, non-fused sfGFP and WalkerV-sfGFP in *E. coli* MCΔFfh. Expression was induced at an OD_600_ of 0.5 for 30 min, the cells were TCA precipitated and loaded on a 12% SDS-PAGE. After Western transfer, immuno detection was carried out with α-GFP and α-rabbit antibodies. Due to induction, a band between 25 and 35 kDa corresponding to the different KdpD-sfGFP fusion proteins and for the non-fused sfGFP was detected.


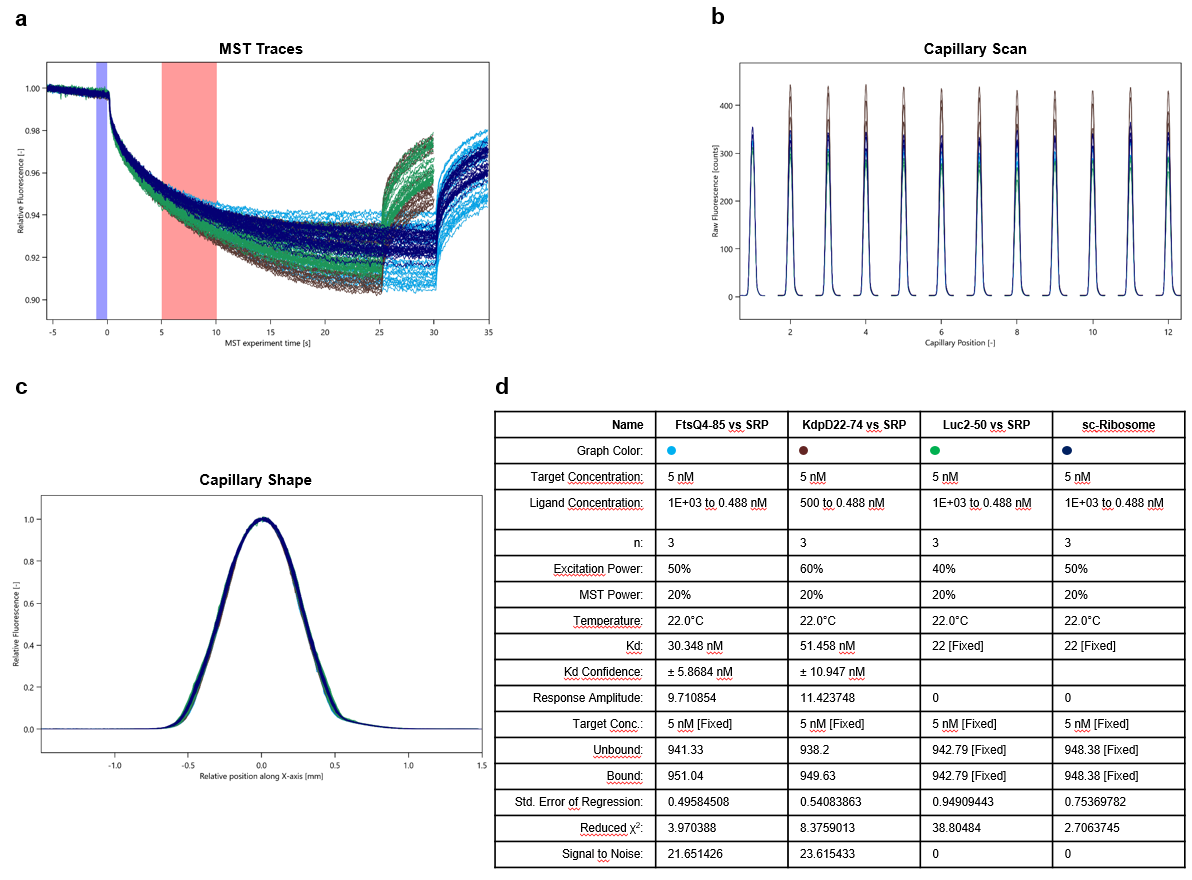
**Figure S4**

**Supplementary Figure S4: Raw data of the MST measurements with FtsQ-4-85, KdpD-22-74 and Luc2-50 as a nascent chain and short-chain (sc)-ribosomes with SRP.**

Microscale thermophoresis measurements of unlabelled SRP (1 µM to 0.49 nM) with labelled RNCs (5 nM) of FtsQ-4-85 (blue), KdpD-22-74 (brown), Luc-2-50 (green) and sc-ribosomes (dark blue). After a 5 min incubation on ice the dilutions were filled into Premium capillaries (NanoTemper Technologies) for the MST measurements. In a, the MST traces (relative fluorescence [-] plotted against the MST experimental time [s]), in b, the capillary scan (raw fluorescence [counts] plotted against the capillary position [-]) and in c, the capillary shape (relative fluorescence [-] plotted against the relative position along x-axis [mm] is shown. In d, the dataset overview is listed.

**Figure S5**


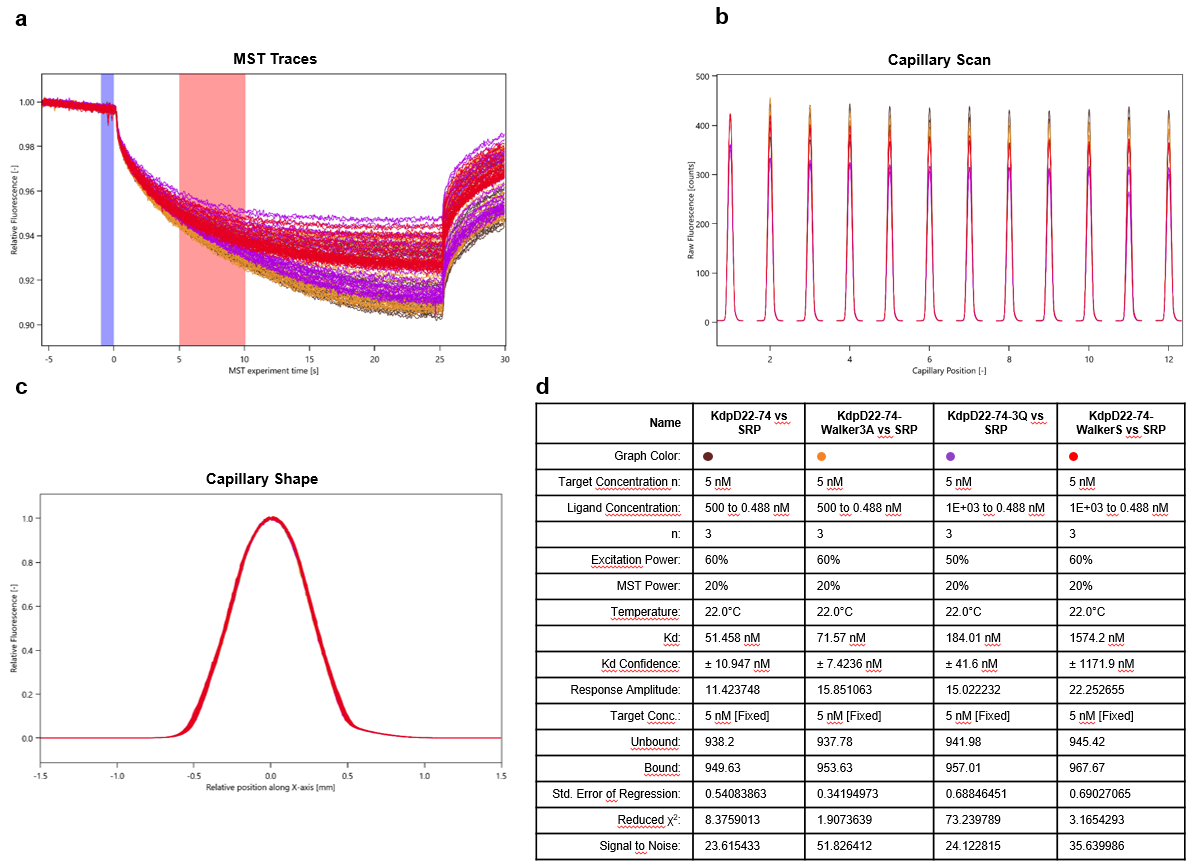


**Supplementary Figure S5: Raw data of the MST measurements with KdpD-22-74, KdpD-22-74-Walker3A, KdpD-22-74-3Q and KdpD-22-74-WalkerS as a nascent chain with SRP.**

Microscale thermophoresis measurements of unlabelled SRP (1 µM to 0.49 nM) with labelled RNCs (5 nM) of KdpD-22-74 (brown), KdpD-22-74-Walker3A (orange), KdpD-22-74-3Q (purple) and KdpD-22-74-WalkerS (red). After a 5 min incubation on ice the dilutions were filled into Premium capillaries (NanoTemper Technologies) for the MST measurements. In a, the MST traces (relative fluorescence [-] plotted against the MST experiment time [s]), in b, the capillary scan (raw fluorescence [counts] plotted against the capillary position [-]) and in c, the capillary shape (relative fluorescence [-] plotted against the relative position along x-axis [mm] is shown. In d, the dataset overview is listed.

**Figure S6**


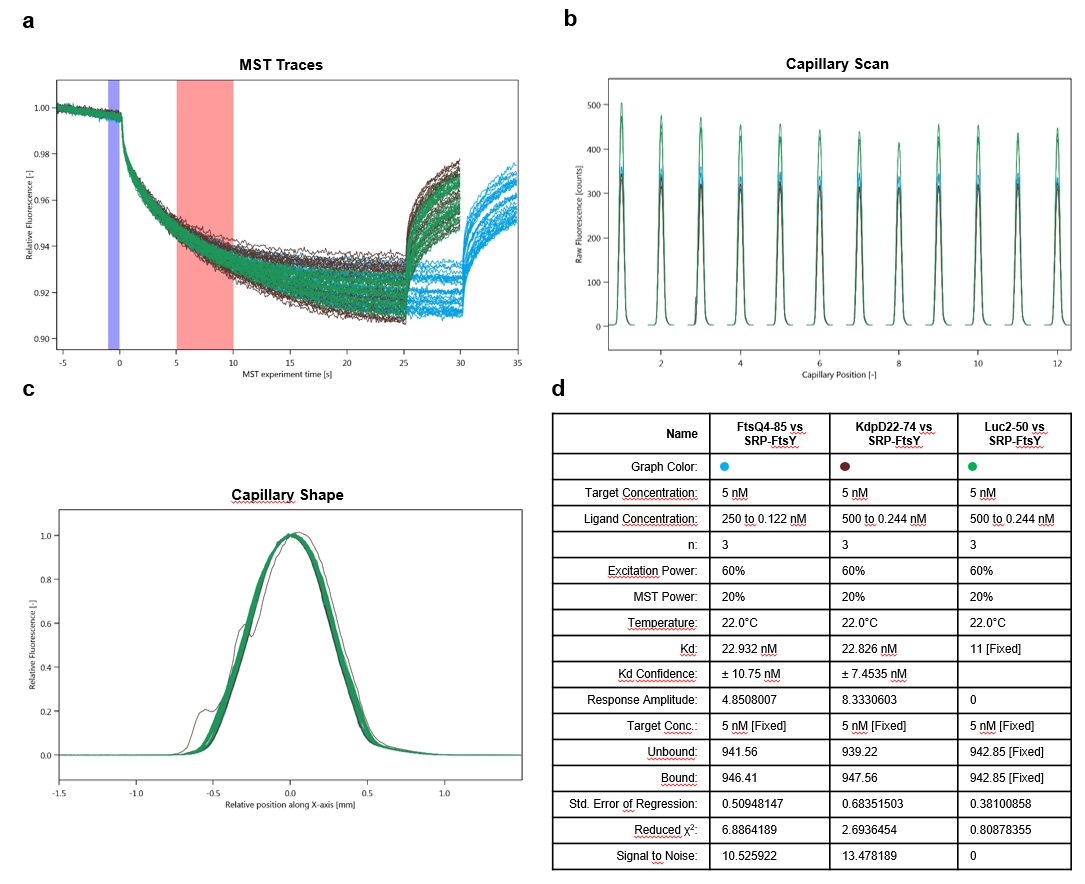


**Supplementary Figure S6: Raw data of the MST measurements with FtsQ-4-85, KdpD-22-74 and Luc2-50 as a nascent chain with a SRP-FtsY complex.**

Microscale thermophoresis measurements of unlabelled SRP-FtsY (500 / 250 nM to 0.244 / 0.122 nM) with labelled RNCs (5 nM) of FtsQ-4-85 (blue), KdpD-22-74 (brown) and Luc-2-50 (green). After a 5 min incubation on ice the dilutions were filled into Premium capillaries (NanoTemper Technologies) for the MST measurements. In a, the MST traces (relative fluorescence [-] plotted against the MST experiment time [s]), in b, the capillary scan (raw fluorescence [counts] plotted against the capillary position [-]) and in c, the capillary shape (relative fluorescence [-] plotted against the relative position along x-axis [mm] is shown. In d, the dataset overview is listed.

**Figure S7**


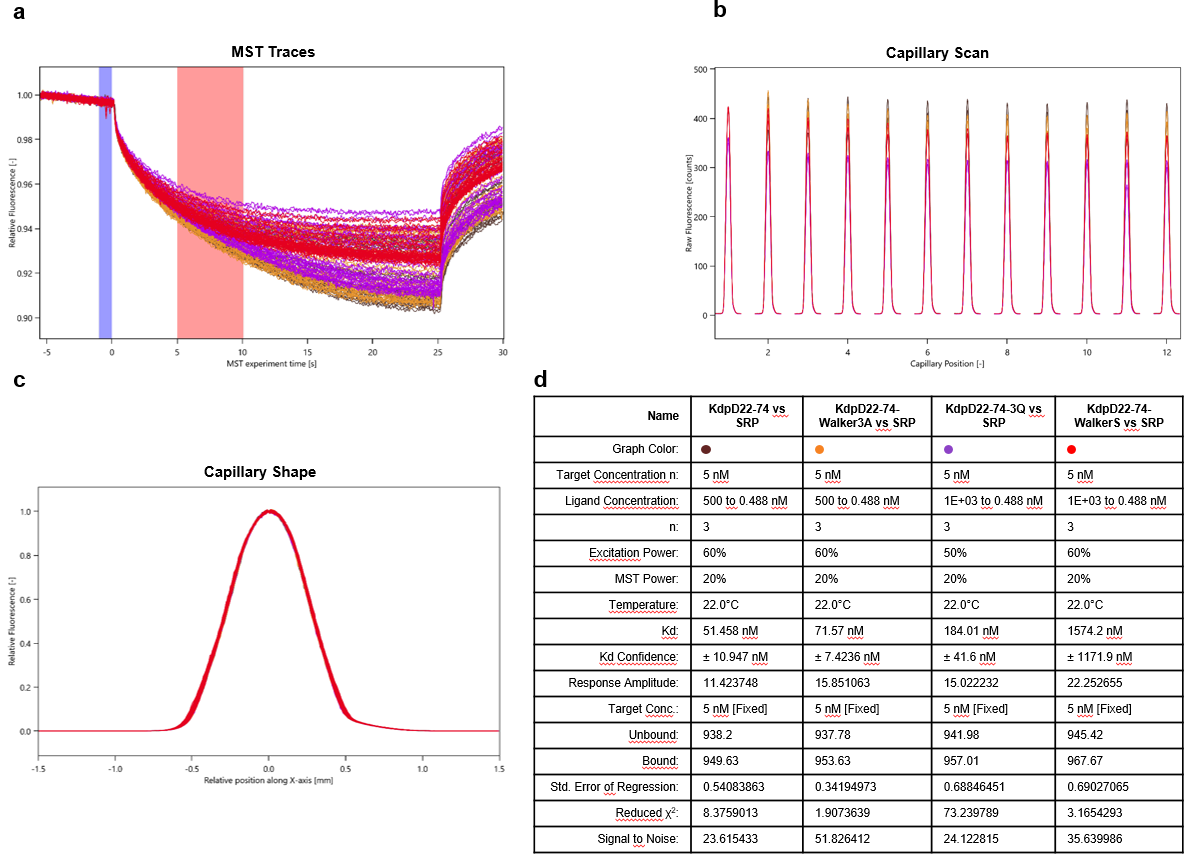


**Supplementary Figure S7: Raw data of the MST measurements with KdpD-22-74, KdpD-22-74-Walker3A, KdpD-22-74-3Q and KdpD-22-74-WalkerS as a nascent chain with a SRP-FtsY complex.**

Microscale thermophoresis measurements of unlabelled SRP (500 nM to 0.244 nM) with labelled RNCs (5 nM) of KdpD-22-74 (brown), KdpD-22-74-Walker3A (orange), KdpD-22-74-3Q (purple) and KdpD-22-74-WalkerS (red). After a 5 min incubation on ice the dilutions were filled into Premium capillaries (NanoTemper Technologies) for the MST measurements. In a, the MST traces (relative fluorescence [-] plotted against the MST experiment time [s]), in b, the capillary scan (raw fluorescence [counts] plotted against the capillary position [-]) and in c, the capillary shape (relative fluorescence [-] plotted against the relative position along x-axis [mm] is shown. In d, the dataset overview is listed.

**
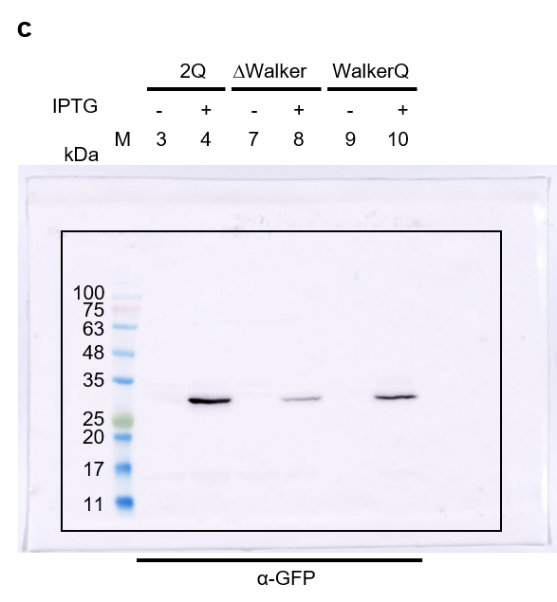

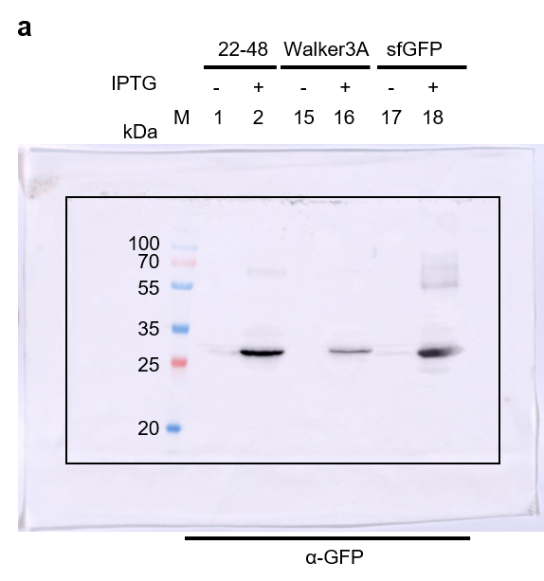

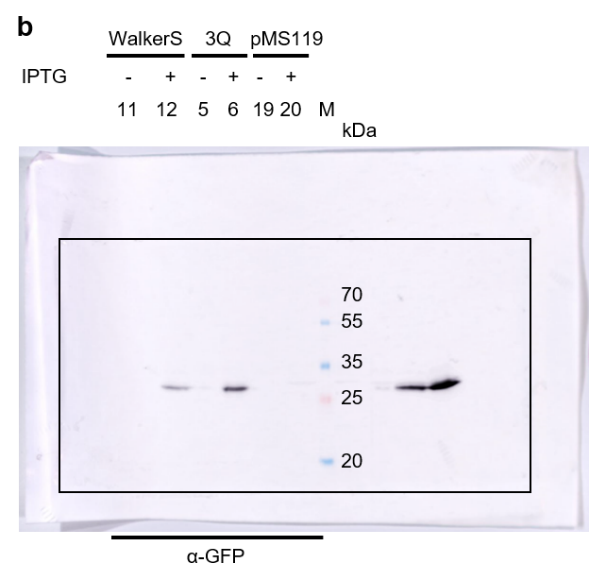
Figure S8**

**
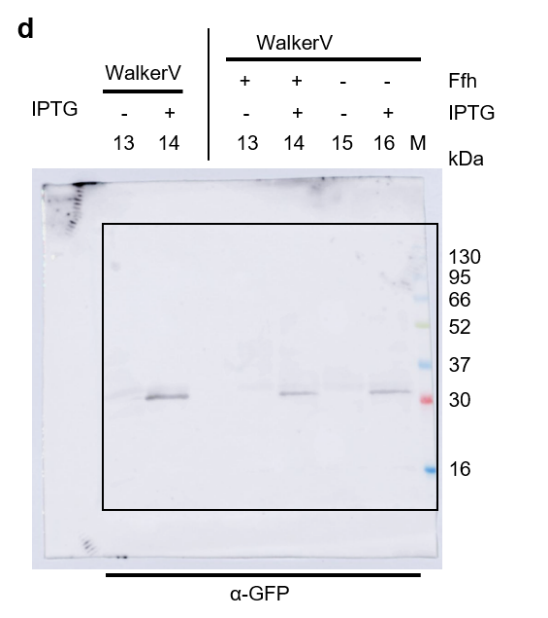

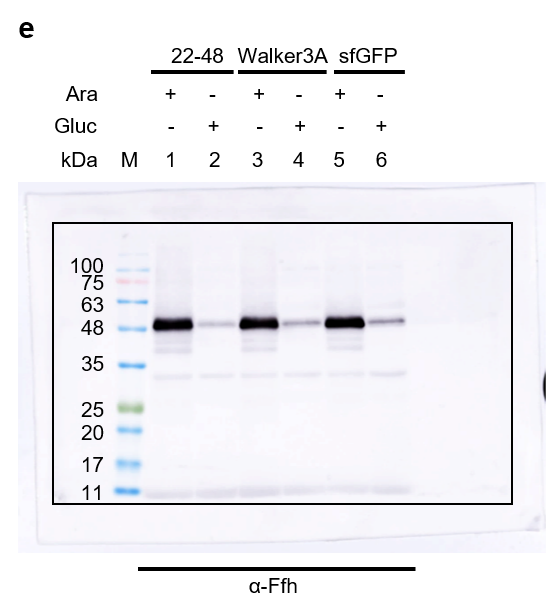

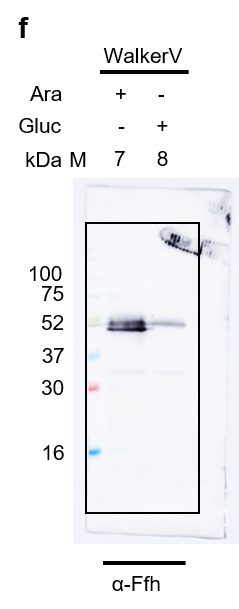
**

**
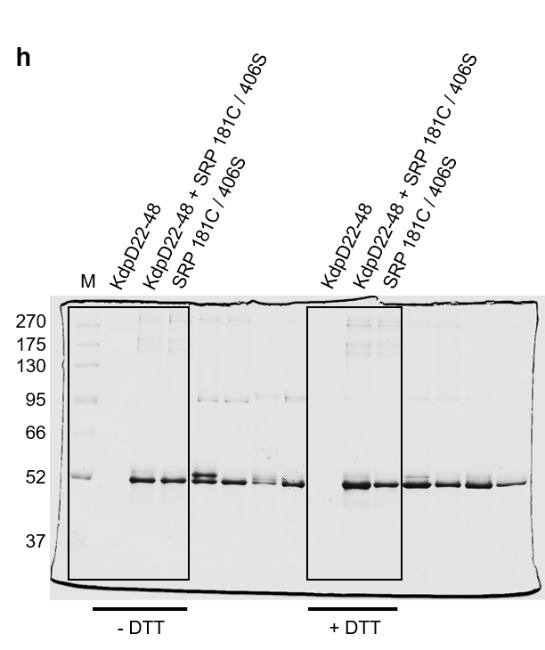
**

**
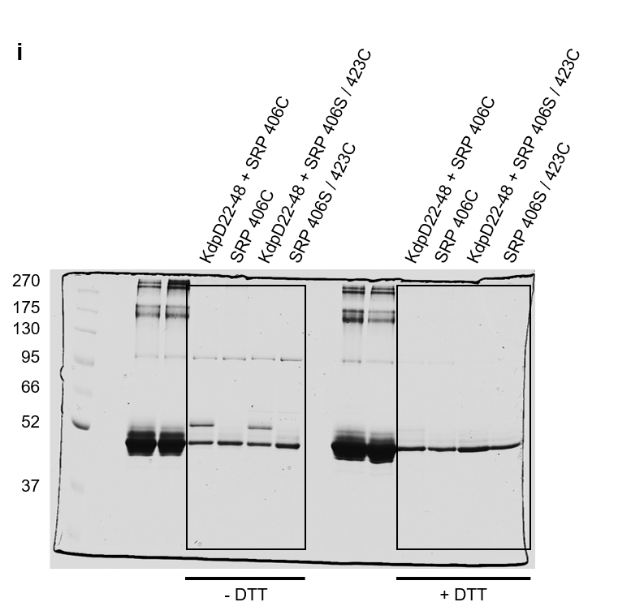
**

**
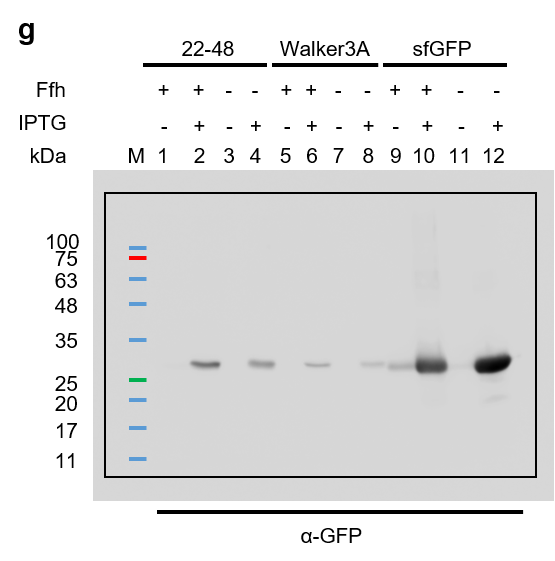
**

**i**

**h**

**Supplementary Figure S8: Uncropped Western Blot and SDS-PAGE of supplementary figure S2 and S3 and of figure 5.** The labelling of the lanes in a, b, c and d corresponds to the labelling in figure S2, the labelling of the lanes in d, e, f and g corresponds to the labelling in figure S3 and the labelling of the lanes in h and i corresponds to figure 5.

**Supplementary Table S1: Oligonucleotides used in this study.**

| Primer name | Sequence (5’ to 3’) | Usage | Plasmid |
| --- | --- | --- | --- |
| KdpD22-48_HindIII | GGCAAGCTTATGCGCGGGAAGCTGAAAG | Amplification | pMS-KdpD22-48-sfGFP |
| KdpD22-48_BamHI | CGGGGATCCCAGTCGCTGGGCTTCTGC |  |  |
| KdpD-R22QK24Q-1 | GATATACATATGCAGGGGCAGCTGAAAGTTTTC | Site-directed mutagenesis | pMS-KdpD22-48-2Q-sfGFP |
| KdpD-R22QK24Q-2 | GAAAACTTTCAGCTGCCCCTGCATATGTATATC |  |  |
| KdpD-R22QK24QK26Q-1 | GCAGGGGCAGCTGCAAGTTTTCTTCGG | Site-directed mutagenesis | pMS-KdpD22-48-3Q-sfGFP |
| KdpD-R22QK24QK26Q-2 | CCGAAGAAAACTTGCAGCTGCCCCTGC |  |  |
| KdpD-K37Q-1 | GCAGGCGTCGGGCAGACCTGGGCGATG | Site-directed mutagenesis | pMS-KdpD22-48-WalkerQ-sfGFP |
| KdpD-K37Q-2 | CATCGCCCAGGTCTGCCCGACGCCTGC |  |  |
| KdpD∆30-38-1 | GCTGAAAGTTTTCTTCTGGGCGATGCTGGCAGAAG | Site-directed mutagenesis | pMS-KdpD22-48-∆Walker-sfGFP |
| KdpD∆30-38-2 | CTTCTGCCAGCATCGCCCAGAAGAAAACTTTCAGC |  |  |
| G30A-G34A-G36A-1 | GTTTTCTTCGCTGCCTGTGCAGCCGTCGCGAAGACCTGGGCG | Site-directed mutagenesis | pMS-KdpD22-48-Walker3A-sfGFP |
| G30A-G34A-G36A-2 | CGCCCAGGTCTTCGCGACGGCTGCACAGGCAGCGAAGAAAAC |  |  |
| KdpD-C32S-1 | CTTCGGTGCCAGCGCAGGCGTCGGG | Site-directed mutagenesis | pMS-KdpD22-48-WalkerS-sfGFP  pMS-KdpD-22-74-C32S-TnaC |
| KdpD-C32S-2 | CCCGACGCCTGCGCTGGCACCGAAG |  |  |
| KdpD-C32V-1 | CTTCGGTGCCGTTGCAGGCGTCG | Site-directed mutagenesis | pMS-KdpD22-48-WalkerV-sfGFP |
| KdpD-C32V-2 | CGACGCCTGCAACGGCACCGAAG |  |  |
| Ffh-L181C-1 | CTGAAAGAAGCCAAATGCAAATTCTACGACGTG | Site-directed mutagenesis | pMS-Ffh-L181C-C-Strep |
| Ffh-L181C-2 | CACGTCGTCGAATTTGCATTTGGC TTCTTTCAG |  |  |
| MscL115-TnaC-MfeI-1 | CTGTATTTTCAGGGACAATTGAGCATTATTAAAGAATTTCG | Site-directed mutagenesis | pMS-MscL^115^ |
| MscL-TnaC-MfeI-2 | CGAAATTCTTTAATAATGCTCAATTGTCCCTGAAAATACAG |  |  |
| MscL-TnaC-NdeI-1 | CCGCACCTGCACCATGGTGTGTGACCTCAAAATGG | Site directed mutagenesis | pMS-MscL^115^ |
| MscL-TnaC-NdeI-2 | CCATTTTGAGGTCACACACCATGGTGCAGGTGCGG |  |  |
| KdpD22-74-MfeI | GGCCAATTGCGGGGGAAGCTGAAAGTTTTC | Amplification | pMS-KdpD-22-74-TnaC  pMS-KdpD-22-74-W3A-TnaC |
| KdpD22-74-NdeI | CGGCCATGGCCCCTCGAGCATGGC |  |  |
| KdpD22-74-3Q-MfeI | GGCCAATTGCAGGGGCAGCTGCAAGTTTTC | Amplification | pMS-KdpD-22-74-3Q-TnaC |
| Luc2-50-EcoRI | GCGAATTCGAAGACGCCAAAAACATAAAG | Amplification | pMS-Luc2-50-TnaC |
| Luc2-50-NdeI | CGGCCATGGGTTCACCTCGATATGTGCATC |  |  |

**Supplementary Table S2: Plasmids used in this study.**

| Plasmids | Name | Characteristics | Reference or source |
| --- | --- | --- | --- |
| pMS119EH |  | Ap^R^, P_tac_, expression vector | Balzer *et al.*, 1992 |
| pMS119EH-sfGFP | sfGFP | Ap^R^, pMS119EH, *sfgfp* | Pross *et al.,* 2016 |
| pMS-KdpD22-48-sfGFP | N22-48-sfGFP | Ap^R^, pMS119EH,  *kdpD*_Δ1-21:Δ49-894_*-sfgfp* | This study |
| pMS-KdpD22-48-2Q-sfGFP | 2Q | Ap^R^, pMS119EH,  *kdpD*_Δ1-21:R22Q,K24Q;Δ49-894_*-sfgfp* | This study |
| pMS-KdpD22-48-3Q-sfGFP | 3Q | Ap^R^, pMS119EH,  *kdpD*_Δ1-21:R22Q,K24Q,K26Q;Δ49-894_*-sfgfp* | This study |
| pMS-KdpD22-48-∆Walker-sfGFP | ∆Walker | Ap^R^, pMS119EH,  *kdpD*_Δ1-21; Δ30-38; Δ49-894_*-sfgfp* | This study |
| pMS-KdpD22-48-WalkerQ-sfGFP | WalkerQ | Ap^R^, pMS119EH,  *kdpD*_Δ1-21:K37Q;Δ49-894_*-sfgfp* | This study |
| pMS-KdpD22-48-WalkerS-sfGFP | WalkerS | Ap^R^, pMS119EH,  *kdpD*_Δ1-21:C32S;Δ49-894_*-sfgfp* | This study |
| pMS-KdpD22-48-Walker3A-sfGFP | Walker3A | Ap^R^, pMS119EH,  *kdpD* _Δ1-21;_ _G30A-G34A-G36A; Δ49-894_ *-sfgfp* | This study |
| pMS-KdpD22-48-WalkerV-sfGFP | WalkerV | Ap^R^, pMS119EH,  *kdpD*_Δ1-21:C32V;Δ49-894_*-sfgfp* | This study |
| pMS-Ffh-C-Strep | Ffh | Ap^R^, pMS119EH, C-terminal Strep tag, *Ffh* | Seitl, 2016 |
| pMS-Ffh-L181C-C-Strep | Ffh 181C / 406S | Ap^R^, pMS119EH, C-terminal Strep tag, *Ffh_L181C, C406S_* | This study |
| pMS-Ffh-M423C-C-Strep | Ffh 406S / 423C | Ap^R^, pMS119EH, C-terminal Strep tag, *Ffh_C406S, M423C_* | Seitl, 2016 |
| pTrc99a-FtsY | FtsY | Ap^R^, pTrc99a, *FtsY*, C-terminal His_6_ tag | Kuhn *et al.,* 2015 |
| pBAT4-MscL^115^ |  | ApR, P_T7_, pBAT4, N-terminal His_6_ and HA tag, TEV site, *mscL_∆116-136_,* TnaC_7-24_ | Seidelt *et al.,* 2009 |
| pMS-MscL^115^ |  | Ap^R^, pMS119EH, N-terminal His_6_ and HA tag, TEV site, *mscL_∆116-136_,* TnaC_7-24_ | This study |
| pMS-KdpD-22-74-TnaC | KdpD-22-74 | Ap^R^, pMS119EH, N-terminal His_6_ and HA tag, TEV site,  *kdpD*_Δ1-21:Δ75-894_, TnaC_7-24_ | This study |
| pMS-KdpD-22-74-W3A-TnaC | KdpD-22-74-W3A | Ap^R^, pMS119EH, N-terminal His_6_ and HA tag, TEV site,  *kdpD* _Δ1-21;_ _G30A-G34A-G36A; Δ75-894_, TnaC_7-24_ | This study |
| pMS-KdpD-22-74-3Q-TnaC | KdpD-22-74-3Q | Ap^R^, pMS119EH, N-terminal His_6_ and HA tag, TEV site,  *kdpD*_Δ1-21:R22Q,K24Q,K26Q;Δ75-894_, TnaC_7-24_ | This study |
| pMS-KdpD-22-74-WalkerS-TnaC | KdpD-22-74-WalkerS | Ap^R^, pMS119EH, N-terminal His_6_ and HA tag, TEV site,  *kdpD*_Δ1-21:C32S;Δ75-894_, TnaC_7-24_ | This study |
| pEM36-3C | FtsQ-4-85 | Ap^R^, N-terminal His_6_ tag, C3-protease cleavage site, *FtsQ_∆1-3; ∆86-276_,* HA-tag, TnaC_7-24_ | Bischoff *et al.,* 2014 |
| pUC19-T7-Luc^50^ |  | Ap^R^, pUC19, N-terminal strep tag, *luc_∆51-550_,* SecM_41-78_, His_6_ tag | Zhang *et al.,* 2010 |
| pMS-Luc2-50-TnaC | Luc2-50 | Ap^R^, pMS119EH, N-terminal His_6_ and HA tag, TEV site,  *luc_∆51-550_*, TnaC_7-24_ |  |
| pMS-His-HA-TnaC | sc-ribosome | Ap^R^, pMS119EH, N-terminal His_6_ and HA tag, TEV site, TnaC_7-24_ | This study |

**References**

Balzer, D., Ziegelin, G., Pansegrau, W., Kruft, V. & Lanka, E. KorB protein of promiscuous plasmid RP4 recognizes inverted sequence repetitions in regions essential for conjugative plasmid transfer. *Nucleic Acids Res*earch **20**, 1851-1858 (1992).

Bischoff, L., Wickles, S., Berninghausen, O., van der Sluis, EO. & Beckmann, R. Visualization of a polytopic membrane protein during SecY-mediated membrane insertion. *Nat Commun.* **5**, 4103 (2014).

Kuhn, P. *et al.* Ribosome binding induces repositioning of the signal recognition particle receptor on the translocon. *J Cell Biol.* **211**, 91-104 (2015).

Pross, E., Soussoula, L. Seitl, I., Lupo, D. & Kuhn, A. Membrane targeting and insertion of the C-tail protein SciP. *J. Mol. Biol*. **428**, 4218-4227 (2016).

Seidelt, B. *et al.* Structural insight into nascent polypeptide chain-mediated translational stalling. *Science* **326**, 1412-1415 (2009).

Seitl, I. Functional and structural studies of C-terminally extended YidC. *Dissertation University of Hohenheim* opus1191 (2016).

Zhang X, Rashid R, Wang K, Shan SO. Sequential checkpoints govern substrate selection during cotranslational protein targeting. *Science* **328**, 757-60 (2010).
